# DExH-Box Helicase 9 Participates in *De Novo* Nrf2 Protein Translation Under Oxidative Stress

**DOI:** 10.1016/j.mcpro.2025.100977

**Published:** 2025-04-23

**Authors:** Wujing Dai, Hongting Diao, Han Qu, Daniel Wurm, Yingying Lu, Qin M. Chen

**Affiliations:** Department of Pharmacy Practice and Science, University of Arizona College of Pharmacy, Tucson, Arizona, USA

**Keywords:** oxidants, antioxidants, stress response, RNA–protein interaction, protein synthesis

## Abstract

Nrf2 transcript factor plays an important role in cellular defense against oxidative stress due to its control for expression of antioxidant and detoxification genes. We have found that Nrf2 gene undergoes *de novo* protein translation when mammalian cells encounter oxidative stress. Here, we report the discovery of DExH-box helicase-9 (DHX9), also known as RNA helicase A, as a binding protein for Nrf2 mRNA at 5′UTR. DHX9 binding to Nrf2 5′UTR increased with increasing doses (50–300 μM) of H_2_O_2_ or treatment time (10–120 min). This incease was in parallel with elevation of Nrf2 protein. Inhibiting DHX9 expression with siRNA or its activity with YK-4-279 inhibitor blocked H_2_O_2_ from inducing Nrf2 mRNA recruitment to the ribosomes or Nrf2 protein elevation. As a nuclear protein, DHX9 was found to increase its abundance in the cytosol with oxidative stress. An increase of DHX9 was detected in the ribosomes from cells treated with H_2_O_2_, most significantly with 100 μM H_2_O_2_, and at 60 min. Ribosomal fractionation revealed an increase of DHX9 protein at 43/48S and 80S fractions in H_2_O_2_-treated cells. H_2_O_2_ treatment caused an RNA-dependent increase of DHX9 interaction with eIF3η. The binding of DHX9 to Nrf2 5′UTR was enhanced by H_2_O_2_-treated cells or by deducting the length of Nrf2 5′UTR. RNase digestion enhanced DHX9 association with the ribosomes. Our data have revealed a novel mechanism of *de novo* Nrf2 protein translation under oxidative stress involving DHX9 binding to Nrf2 5′UTR, perhaps *via* removal of a negative RNA element, to recruit 43S preinitiation complex for translation initiation.

## Highlights

• Little is known about how certain proteins can be translated under stress conditions.
• DHX9 was discovered as a binding protein for Nrf2 5’UTR under oxidative stress.
• DHX9 interacts with 43S pre-initiation complex for Nrf2 protein translation.
• RNA structural remodeling participates in translation regulation under stress conditions.

## Graphical Abstract

**Figure:**
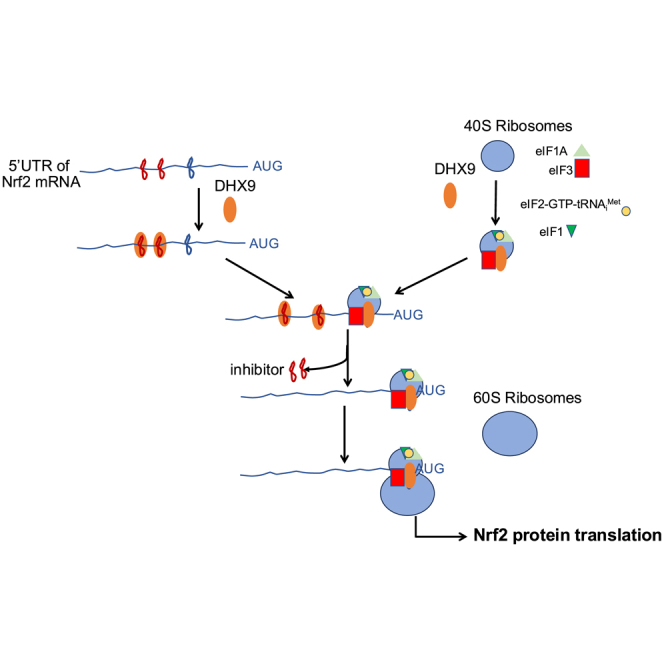

## In Brief

Little is known for how proteins are translated under stress conditions. LC-MS/MS–based proteomics led to the discovery of DHX9 as a binding protein for Nrf2 mRNA under oxidative stress. DHX9 interacts with Nrf2 5′UTR and 43S preinitiation complex for translation initiation of Nrf2 protein. As an RNA helicase, DHX9 may remove inhibitory elements to allow eIFs and translation machinery to carry out translation of Nrf2 protein under oxidative stress conditions.

Mammalian cells respond to physical and chemical stress by turning off protein synthesis in general. Evolutionarily, this serves to prevent aberrant protein synthesis and reduces the burden for cells to deal with during stress. Since over 30% ATP generated in a cell is utilized for protein synthesis, this shutdown mechanism conserves energy for effective dealing of cellular stress (1, 2, 3). There are about 100 genes that have been reported capable of bypassing such general protein synthesis inhibition and being translated selectively under stress conditions. Examples of these genes include c-Myc, HIF-1α, FGF2, VEGF, Apaf1, XIAP, bcl-2, bcl-xL, Bag1, and BiP (4, 5, 6). We have reported that Nrf2 gene undergoes oxidative stress–induced protein translation (7, 8, 9, 10, 11, 12, 13). Since Nrf2 encodes a transcription factor regulating antioxidant and detoxification response, it is important to understand how Nrf2 escapes the general protein translation control and becomes translated selectively.

The process of protein translation is divided into three sequential stages: initiation, elongation and termination, with the initiation being a rate-limiting step. Normally, translation initiation requires 5′ m^7^G cap of mRNA for recognition by eukaryotic initiation factor 4E (eIF4E), a 3′ poly(A) RNA tail for poly (A)-binding protein (PABP), and participation of at least 12 eIFs (14). During stress, this 5′ m^7^G cap-dependent translation is inhibited. Instead, the translation machinery recognizes the internal ribosomal entry site (IRES) in the 5′UTR of the mRNA species. This is facilitated by the IRES trans-acting factors (ITAFs) for translation initiation (6, 15, 16). IRES-mediated protein translation was first discovered from viral infection, when cellular protein synthesis is inhibited (17). A few ITAFs have been characterized, with some mediate viral protein translation as well as cellular protein translation under stress (16, 18). These ITAFs presumably recruit eIFs and 43S preinitiation complex containing the small subunit of ribosomes for translation initiation.

Proteomic techniques provide a useful tool for discovery of RNA-binding proteins. We have found that 5′UTR of Nrf2 mRNA increases binding to La autoantigen, elongation factor 1a (EF1a) and far upstream binding protein 1 (FUBP1) for regulating Nrf2 protein translation when cells encounter oxidative stress (8, 9, 10, 11). La undergoes nuclear export, increases it’s binding to Nrf2 5′UTR and is essential for oxidant-induced Nrf2 protein translation. Unlike La, EF1a binds to a small area, 27 nucleotides, of Nrf2 5′UTR containing a G-quadruplex structure (10). The C-terminal region of EF1a contains several arginine-glycine-glycine motifs (19), providing the structural feature for G-quadruplex binding. EF1a does not change subcellular localization or association with ribosomes during oxidative stress, arguing against its role in coordinating the translational machinery involving eIFs (10). In contrast, FUBP1 increases binding to Nrf2 5′UTR and interacts with eIF3 for translation initiation of Nrf2 (11). Despite of these discoveries, how Nrf2 undergoes *de novo* protein translation under stress conditions remains to be fully understood.

The proteomic technology has evolved over time. Improvements of the instrument in sensitivity and versatility provide the opportunity for identification of additional proteins. Using a shotgun approach and improved LC-MS/MS instrument from our previous studies, we addressed whether or not there are additional proteins capable of interacting with the 5′UTR of Nrf2 mRNA for oxidant induced Nrf2 protein translation. Here, we report the discovery of DHX9 in mediating Nrf2 protein translation when cells encounter oxidative stress.

## Experimental Procedures

### Cell Culture, H_2_O_2_ Treatment, and Reagents

HeLa cells (CCL-2) were obtained from ATCC and cultured in Dulbecco's modified Eagle's medium (DMEM, 10013CV, Corning) with 10% fetal bovine serum (FBS, S11150H, R&D Systems), 100 U/ml penicillin, and 100 μg/ml streptomycin (15140122, Gibco). Cells were subcultured every 2 to 3 days. For experiments, cells were seeded in 6-well, 24-well, or 100 mm culture dishes. When reaching 80% confluency, cells were placed in DMEM containing 0.5% FBS for 16 to 20 h under the culture condition for serum starvation. Cells were treated with H_2_O_2_ for 10 min to induce oxidative stress followed by removal of oxidized medium and culture in fresh DMEM with 0.5% FBS for indicated time periods. When harvest time was 1 h, culture medium was not changed, since our data showed no difference with *versus* without medium change. All reagents were purchased from Sigma-Aldrich, except DHX9 inhibitor YK-4-279 (A11612), which was obtained from AdooQ BioScience.

### *In Vitro* Transcription

Nrf2 5′UTR sequence (NM_006164.3) was cloned into pJet hsa plasmid as described (8). To generate the full length or fragments of Nrf2 5′UTR, 10 ng of the plasmid DNA was used as a template for PCR with: forward primer containing T7 polymerase binding site: 5′-TAATACGACTCACTATAGGGAAATCAGGGAGGCGCAGCTC-3′; and reverse primers for Nrf2 5′UTR of −555 to 0 nt: 5′-GATGAGCTGTGGACCGTGTG-3′; −555 to −225 nt fragment: 5′-GGCAAGAGTCCGGGCCCTTC-3′; −555 to −333 nt fragment: 5′-AAGGGACTGCCAGCTGGGGT-3′; or −555 to −450 nt fragment: 5′-CGAGATAAAGAGTTGTTTGC-3′. The PCR products were purified by agarose gel electrophoresis and validated by sequencing, before serving as a template (1 μg) for *in vitro* transcription with a TranscriptAid T7 High Yield Transcription Kit (K0441, Thermo Fisher Scientific) in the presence of Biotin-16-UTP (11388908910, Roche) for 2-h incubation at 37 °C. The synthesized RNAs were extracted by RNeasy Mini Kit (74104, Qiagen).

### RNA Affinity Chromatography for Identification of RNA-Binding Proteins

Cells were harvested in a nucleic acid binding buffer [10 mM Hepes (pH 7.6), 5 mM MgCl_2_, 40 mM KCl, 1 mM DTT, 5% glycerol, and 5 mg/ml heparin] containing 1× protease inhibitors from a cocktail (P8340, Sigma-Aldrich) and lysed by sonication 3 times for 5 s each on ice. Cell lysates were centrifuged at 18,000*g* at 4 °C for collecting the supernatants for *in vitro* RNA-binding reaction (8, 20). The supernatant containing 500 μg of proteins was incubated with 5 μg of biotinylated Nrf2 5′UTR RNA probes on ice for 1 h, followed by addition of 0.2 ml of Streptavidin Sepharose beads (17,511,301, GE Healthcare), which were pre-equilibrated with the binding buffer. After overnight incubation with rotation at 4 °C, the beads were loaded on a 2 ml column (89896, Pierce) and washed 3 times with 2 ml of 1 M NaCl in the nucleic acid binding buffer by gravity. The captured proteins were released by boiling in water for LC-MS/MS analysis or were boiled in SDS-PAGE loading buffer for 10% SDS-PAGE.

### Mass Spectrometry, Experimental Design, and Statistical Rationale for Proteomic Data Analysis

Samples were prepared from three independent experiments (n = 3) for binding to RNA affinity columns made from *in vitro* synthesized Nrf2 5′UTR, as described above. Each experiment contained one pair of RNA affinity chromatography elutes from control untreated and H_2_O_2_-treated cells. The choice of three pairs of samples from control and H_2_O_2_-treated cells was based on the success rate from our previous works using proteomic technologies (8, 10, 21, 22). The samples were subjected to overnight digestion with Trypsin (20 μg/ml) and ZipTip sample clean up according to the manufacturer’s instructions (Millipore). The analyses were performed by a Linear Trap Quadrupole Orbitrap Velos mass spectrometer (Thermo Fisher Scientific) equipped with an Advion nanomate ESI source (Advion). The instrument has daily quality control using HeLa cell lysates prior to running experimental samples.

Peptides were eluted from a C18 precolumn (100-μm id × 2 cm, Thermo Fisher Scientific) onto an analytical column (75-μm ID × 10 cm, C18, Thermo Fisher Scientific) using a beginning and holding at 2% solvent B (0.1% formic acid in acetonitrile) for 5 min, followed by 2 to 7% gradient of solvent B over 5 min, followed by a 7 to 15% gradient of solvent B over 50 min, a 15 to 35% gradient of solvent B over 60 min, a 35 to 40% gradient of solvent B over 28 min, a 40 to 85% gradient of solvent B over 5 min, held at 85% solvent B for 10 min, 85-2% gradient of solvent B for 1 min then held at 2% solvent B for 16 min. All flow rates were at 400 nl/min, with solvent A being 0.1% formic acid in water.

Data-dependent scanning was performed by the Xcalibur v 2.1.0 software (https://tools.thermofisher.com/content/sfs/manuals/Man-XCALI-97212-Xcalibur-21-Quan-ManXCALI97212-E-EN) (23) using a survey mass scan at 60,000 resolution in the Orbitrap analyzer scanning *m/z* 400 to 1600, followed by collision-induced dissociation tandem mass spectrometry (MS/MS) of the 14 most intense ions in the linear ion trap analyzer. Precursor ions were selected by the monoisotopic precursor selection setting with selection or rejection of ions held to a ± 10 ppm window. Dynamic exclusion was set to place any selected *m/z* on an exclusion list for 45 s after a single MS/MS. All MS/MS spectra were searched against the Human database downloaded from Human_UniprotProteome_2015_1006_cont.fasta using Thermo Proteome Discoverer 1.3 (Thermo Fisher Scientific; https://assets.thermofisher.com/TFS-Assets/CMD/manuals/Man-XCALI-97358-Proteome-Discoverer-13-User-ManXCALI97358-A-EN). This database contained 69,691 proteins. Fully tryptic peptides with up to two missed cleavage sites were selected, with fragment tolerance of 0.80 Da (monoisotopic), precursor tolerance of 10.0 PPM (monoisotopic), and no fixed modifications. Variable modifications considered during the search included methionine oxidation (15.995 Da) and cysteine carbamidomethylation (57.021 Da). No contaminants were excluded during search.

Proteins were identified at 99% confidence with peptide threshold of 95% using XCorr score cutoffs as determined by a reversed database search (24). The results were also validated using X!Tandem (https://www.thegpm.org/tandem), another search engine, and displayed with Scaffold v 3.6.1 (Proteome Software Inc; https://www.proteomesoftware.com), a program relying on various search engine results (*i.e.* Sequest, X!Tandem, MASCOT) and using Bayesian statistics to reliably identify spectra (25). The estimated false discovery rate for detected peptides was 0.00%. The number of unique peptides assigned to each protein and percentage of protein sequence coverage are indicated in supplemental Table S1. The raw data are available *via* ProteomeXchange with identifier PXD055838.

To determine the differences between control and H_2_O_2_-treated cells, descriptive statistics, including means and SD, were computed from quantitative value (normalized total spectra) of each identified protein (ID) when Keratins and those containing 0 value in any of the experimental groups were removed (supplemental Table S2). A paired *t* test was performed for each ID to determine whether the means between control and treatment conditions were statistically significant. The paired *t* test was chosen because measurements for each ID were obtained from the same experimental and technical conditions for each pair of control and H_2_O_2_-treated samples. This approach accounts for intrasubject variability and improves statistical power by reducing interexperimental variations. The significance was determined at *p* < 0.05 (supplemental Table S2).

### Far Western Blot

HeLa cells grown in 100 mm dishes were treated with H_2_O_2_ and lysed in 400 μl polysome lysis buffer [20 mM Tris–HCl (pH 7.5), 100 mM KCl, 5 mM MgCl_2_, 0.5% Nonidet P-40] containing 1 mM DTT and 1× protease inhibitors from a cocktail (P8340, Sigma-Aldrich). The cytosolic fraction collected from 18,000×*g* centrifugation (300 μl) was mixed with 100 μl 10× protein-RNA binding buffer [0.2 M Tris (pH 7.5), 0.5 M NaCl, 20 mM MgCl_2_, 1% Tween-20], 300 μl 50% glycerol, and 300 μl nuclease-free water containing 100 U RNase inhibitor and 50 pmol of biotinylated RNA probe. The mixture was incubated overnight at 4 °C with rotation before 30 μl streptavidin agarose beads (20349, Thermo Fisher Scientific) were added for another 4-h incubation with rotation at 4 °C. The beads were washed 4 times with 1× wash buffer [20 mM Tris (pH 7.5), 10 mM NaCl, 0.1% Tween-20] before 50 μl 1× Laemmli buffer (2% SDS, 10% glycerol, 62.5 mM Tris-Cl, pH 6.8, 0.002% bromphenol blue, 5% 2-mercaptoethanol) was added to the beads for boiling. The eluted proteins were then analyzed by Western blot.

### RNA–Protein Complex Immunoprecipitation

HeLa cells were lysed on ice with polysome lysis buffer containing 100 U/ml RNase inhibitor (EO0384, Thermo Fisher Scientific), 1 mM DTT and 1× protease inhibitors. The cell lysate was centrifuged at 18,000*g* at 4 °C for 10 min to collect the supernatant. After preclearing with protein A/G magnetic agarose beads (78609, Thermo Fisher Scientific) by 1 h incubation with rotation, the cytosolic fraction was incubated overnight at 4 °C with 1 μg DHX9 antibody (sc-137232) or mouse IgG (sc-2025, Santa Cruz Biotechnology). The protein A/G beads were added to the mixture for 4-h incubation at 4 °C with rotation. After three washes with the polysome lysis buffer, the bound RNA was extracted with Trizol (15596018, Invitrogen) from the beads for detection of Nrf2 mRNA by RT-quantitative PCR (qPCR).

### RT-qPCR Analysis

RNA was reverse transcribed to cDNA using a qScript cDNA SuperMix kit (95048, QuantaBio) for subsequent quantitative PCR (qPCR). The qPCRs were performed using the primer pairs of Nrf2 forward 5′-TTCCCGGTCACATCGAGAG-3′, Nrf2 reverse 5′-TCCTGTTGCATACCGTCTAAATC-3′; and GAPDH forward 5′- GGAGCGAGATCCCTCCAAAAT-3′, GAPDH reverse 5′- GGCTGTTGTCATACTTCTCATGG-3′. The qPCR was performed with PowerUp SYBR Green (A25777, Applied Biosystems) on a Bio-Rad CFX96 thermal cycler, with Nrf2 mRNA abundance calculated with Bio-Rad CFX Manager Software (https://www.bio-rad.com/en-us/sku/1845000-cfx-manager-software).

### siRNA Transfection

Subconfluent HeLa cells in 12-well plates were transfected with either control siRNA (sc-37007, Santa Cruz Technology) or DHX9 siRNA (sc-45706, Santa Cruz Technology) following the manufacturer’s protocol. For each well, cells were exposed to 40 pmol siRNA and 4 μl Lipofectamine 3000 in 400 μl Opti-MEM (51985034, Gibco) for 6 h. Without removing the transfection mixture, 500 μl DMEM containing 20% FBS was added for incubation of an additional 16 h under cell culture conditions. The cells were then placed in 1 ml DMEM containing 10% FBS and cultured for 32 h before 16 h serum starvation, followed by H_2_O_2_ treatment.

### Western Blot and Subcellular Fractionation

For total cell lysates, HeLa cells in culture dishes were dissolved in 1×Laemmli sample buffer and subject to SDS-PAGE. For collecting cytosol *versus* nuclear enriched fractions, HeLa cells were scrapped in ice-cold PBS and span down by centrifugation at 500*g* for 5 min. The cell pellets were triturated by repetitive pipetting in the polysome lysis buffer containing protease inhibitors. Upon centrifugation at 1,000*g* for 5 min, the supernatant was collected as the cytosolic fraction and the pellet was treated as the nuclear enriched fraction. The pellets were washed once by resuspension in the polysome lysis buffer and centrifugation at 1,000*g*. After denaturing the cytosolic or nuclear fractions in the Laemmli sample buffer by boiling and sonicating, the samples from equal cell number or equal protein concentration were loaded for SDS-PAGE. After separation by electrophoresis, the proteins were transferred to a polyvinylidene difluoride membrane using a Bio-Rad Trans-Blot Turbo Transfer System. The membranes were blotted with following: Nrf2 antibody (sc-13032), DHX9 antibody (sc-137232, sc-137183), GAPDH antibody (sc-32233), Lamin B1 antibody (sc-377000), S6 antibody (sc-74459), L36a antibody (sc-100831), eIF4E antibody (sc-9976), eIF3η antibody (sc-16377), eIF2α antibody (sc-133132), or eIF1 antibody (sc-390122). The bound antibodies were recognized by anti-mouse IgG-HRP (A9044), anti-rabbit IgG-HRP (A9169), or anti-goat IgG-HRP (sc-2354). All antibodies were obtained from Santa Cruz Biotechnology, except anti-mouse or anti-rabbit secondary antibodies, which were purchased from Millipore Sigma-Aldrich.

### Immunocytochemistry

HeLa cells were cultured on 0.13 to 0.17 mm thick circle cover glasses in 24-well plates and treated with H_2_O_2_ when the confluence is 50%. To identify subcellular DHX9 localization, HeLa cells were fixed in 4% paraformaldehyde for 10 min before incubation with 0.2% Triton X-100 to permeabilize the plasma membrane and the membrane of subcellular organelles. To permeabilize the plasma membrane only for staining cytosolic DHX9, following 4% paraformaldehyde fixation, cells were incubated with 0.5% saponin for 10 min at room temperature. Nonspecific binding was blocked by 2% bovine serum albumin in PBS containing 0.1% Tween-20 without (for total DHX9) or with 0.1% saponin (for cytosolic DHX9) for 1 h at room temperature. The cells were incubated overnight at 4 °C with DHX9 antibody (1: 50 dilution, sc-137232; Santa Cruz Biotechnology) or Phospho-S6 antibody (1: 200 dilution, #4858, Cell Signaling Technology). Alexa Fluor 488–conjugated Chicken anti-Mouse IgG (Invitrogen, A21200) or Alexa Fluor 568–conjugated Donkey anti-Rabbit IgG (Invitrogen, A10042) was used as the secondary antibody at a 1:2000 dilution. The slides were washed by PBS containing 0.1% Tween-20 without (for total DHX9) or with 0.1% saponin (for cytosolic DHX9). ProLong Gold Antifade Mountant containing 4′,6-diamidino-2-phenylindole (Invitrogen, P36931) was used to mount the cover glasses. Images were captured under 63× objective with a Marianas system (3i Biotechnology).

### Isolation of Ribosomes

HeLa cells were treated with 100 μg/ml cycloheximide (CHX) for 10 min at 37 °C before harvesting. After two washes in ice cold PBS containing 100 μg/ml CHX, cells were scrapped off in the same buffer. Cell pellets were collected by spinning down at 500*g* for 5 min and resuspended in 1 ml polysome lysis buffer [20 mM Tris–HCl (pH 7.5), 100 mM KCl, 5 mM MgCl2, 0.5% IGEPAL CA-630] containing freshly added 1× protease inhibitor (EDTA-free), 100 μg/ml CHX, 1 mM DTT, 25 μM MG132, and 100 U/ml RNase Inhibitor. After incubating on ice for 10 min with mixing by inversion every 2 min, cell debris, nuclei, and mitochondria were removed by centrifuging at 12,000×*g* for 10 min. The supernatant was transferred onto the top of 10% + 35% sucrose cushion and centrifuged at 32,000 rpm with SW41 rotor for 2 h at 4 °C. Ribosomal pellets were collected at the bottom and resuspended in Trizol or 1×Laemmli sample buffer for RT-qPCR or Western Blot analysis.

For ribosomal fractionation, the cytosolic fraction was loaded onto a linear 10 to 50% w/v sucrose gradient and centrifuged at 39,000 rpm with a SW41 rotor for 2 h at 4 °C. The gradient was displaced upright with 60% sucrose and the distribution of ribosomal subunits was recorded at A 254 nm with a BioLogic Liquid Chromatography System (Bio-Rad). The fractions were collected every 30 s at a flow rate of 1 ml/min. Each collected fraction was subjected to 2× volume 100% ethanol for overnight precipitation at −20 °C. The precipitated proteins were dissolved in 1× Laemmli buffer for Western blot analysis.

### Immunoprecipitation for Protein–Protein Interaction

HeLa cells were lysed in radioimmunoprecipitation (RIPA) buffer (1% Triton X-100, 140 mM NaCl, 0.1% SDS, 0.1% sodium deoxycholate, 10 mM Tris, pH 8.0) containing 1 mM DTT, and 1× protease inhibitors. For RNase digestion, a final concentration of 10 U/ml RNase A/T1 mix (Thermo Fisher Scientific, EN0551) or 100 U/ml RNase inhibitor (Thermo Fisher Scientific, EO0382) was added to RIPA buffer. After removal of the precipitates by centrifugation at 18,000*g* for 15 min at 4 °C, the supernatant was precleared with 20 μl protein A/G agarose beads by 1 h incubation, followed by overnight incubation at 4 °C with 1 μg DHX9 antibody (sc-137232) or mouse IgG (sc-2025; Santa Cruz Technology). After mixing with 30 μl protein A/G agarose beads for 4-h incubation with rotation at 4 °C, the antibody–protein complex was captured by centrifugation. After three washes with RIPA buffer, proteins were eluted by 1×Laemmli sample buffer for Western blot analysis.

### Statistical Analyses

Statistical analyses for non–mass spectrometry data were performed by GraphPad Prism 9 software (https://www/graphpad.com). Data with one grouping variable were analyzed with one-way ANOVA and corrected by Dunnett‘s multiple comparisons test. Means of two groups were compared by two-tailed *t* test. *p* value < 0.05 was set as the threshold for significant difference.

## Results

### DHX9 Interacts with Nrf2 mRNA under Oxidative Stress Condition

**Fig. 1:**
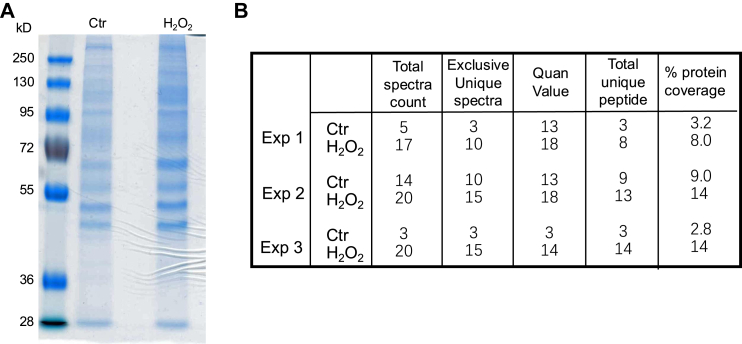
**Evidence of protein binding to Nrf2 5′UTR due to H_2_O_2_-induced oxidative stress.** Biotinylated RNA containing Nrf2 5′UTR sequence was incubated with cell lysates extracted from 1 h 100 μM H_2_O_2_-treated HeLa cells. The bound proteins were isolated and analyzed by SDS-PAGE or LC-MS/MS. The image of Coomassie blue-stained SDS polyacrylamide gel from one experiment representative of three independent experiments is shown (*A*). Shotgun approach of LC-MS/MS analyses of Nrf2 5′UTR bound proteins revealed the presence of DHX9, with the numbers of total spectra, exclusive unique spectra, quantitative value (Quan value, normalized total spectra), total unique peptide, and the percentage of coverage of DHX9 protein shown (*B*). DHX9, DExH-box helicase-9.

We have found that oxidants induce *de novo* Nrf2 protein translation and 5’UTR of Nrf2 mRNA plays a role (8, 9, 10, 11, 26). Using *in vitro* synthesized biotinylated RNA containing Nrf2 5′UTR sequence as a bait, we found that the lysates from H_2_O_2_-treated cells gained binding of multiple proteins to Nrf2 5′UTR (Fig. 1*A*). The binding proteins were identified using a shotgun proteomic approach. With three independent batches of control and H_2_O_2_-treated cells, the proteins bound to Nrf2 5′UTR bait were analyzed by a Linear Trap Quadrupole mass spectrometer. Statistical analyses revealed that ATP-dependent RNA helicase A (Q08211, DHX9) had a significant increase, with quantitative value (normalized total spectra) of 9.14 ± 5.34 for control or 16.53 ± 2.54 for H_2_O_2_-treated cells (*p* < 0.05). The numbers for total spectra count, exclusive unique spectra, quantitative value, total unique peptide or percentage of coverage of DHX9 protein detected are shown from three independent experiments (Fig. 1*B*). These numbers suggested an increased abundance of DHX9 protein for binding to Nrf2 5′UTR in the stressed cells.

**Fig. 2:**
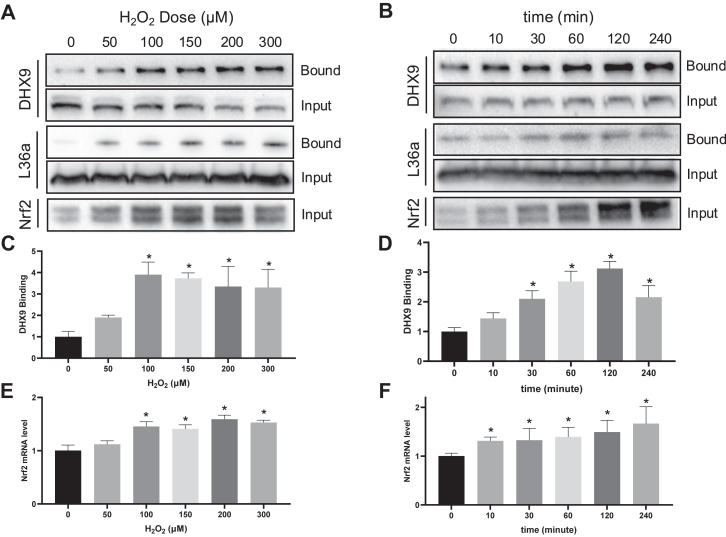
**H_2_O_2_ causes a dose- and time-dependent increase of DHX9 binding to Nrf2 mRNA.** HeLa cells were treated with indicated doses of H_2_O_2_ (*A*, *C*, and *E*) and collected at 1 h or with 100 μM H_2_O_2_ for 10 min and collected at indicated time points (*B*, *D*, and *F*). For Far Western (*A*–*D*), cells were lysed and incubated with 5 μg biotinylated Nrf2 5′UTR RNA for isolation of bound DHX9 or L36a for analysis by Western blot. The results from one representative experiment are shown (*A* and *B*). The band intensities were quantified by ImageJ and shown as means ± SD from three independent experiments (*C* and *D*). For RNP-IP (*E* and *F*), DHX9 was immunoprecipitated for RNA extraction by Trizol and RT-qPCR to detect Nrf2 mRNA. The data represent means ± SD from three independent experiments (*E* and *F*). ∗ indicates statistical significance (*p* < 0.05) compared to the 0 μM H_2_O_2_ (*C* and *E*) or 0 min treatment group (*D* and *F*) by one-way ANOVA. DHX9, DExH-box helicase-9; qPCR, quantitative PCR; RNA-IP, ribonucleoprotein immunoprecipitation.

We validated DHX9 binding to Nrf2 5′UTR by Far Western blot using biotinylated Nrf2 5′UTR as a bait. H_2_O_2_ treatment caused a dose-dependent increase of DHX9 binding to Nrf2 5′UTR, correlating with Nrf2 protein elevation (Fig. 2, *A* and *C*). The time-course studies revealed that DHX9 increased its interaction with Nrf2 5′UTR at 30 min after H_2_O_2_ treatment (Fig. 2, *B* and *D*). The ribosomal protein L36a was included as a positive control, showing that DHX9 interaction with Nrf2 5′UTR occurred in parallel with if not preceded L36a interaction with Nrf2 5′UTR (Fig. 2, *B* and *D*).

To demonstrate the interaction between DHX9 and Nrf2 mRNA *in cellulo* during oxidative stress, we performed ribonucleoprotein immunoprecipitation (IP). In this assay, the cytosolic DHX9 was isolated by IP for detection of Nrf2 mRNA by RT-qPCR. The dose-response and time-course experiments showed that H_2_O_2_ induced increases of Nrf2 mRNA in the DHX9 IP complexes (Fig. 2, *E* and *F*).

### DHX9 Mediates Nrf2 Protein Induction under Oxidative Stress

**Fig. 3:**
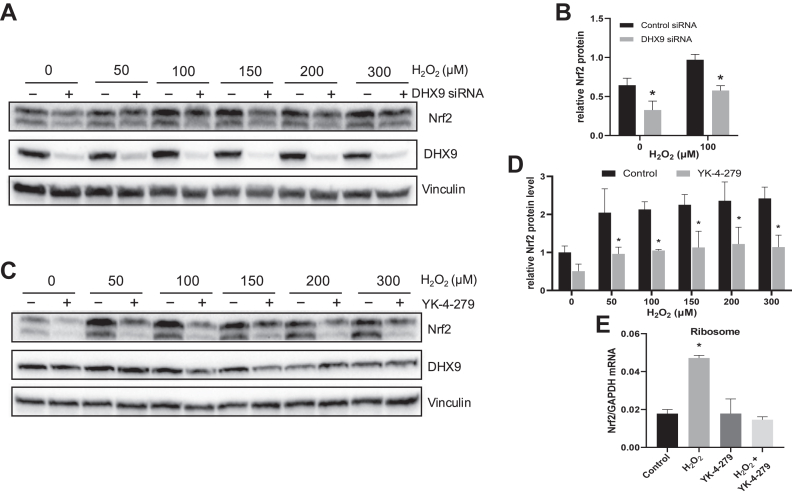
**Inhibition of DHX9 blocks H_2_O_2_ from inducing Nrf2.** HeLa cells were transfected with control or DHX9 siRNA for 48 h (*A* and *B*) or pretreated with 1 μM YK-4-279 for 16 h (*C*–*E*) before treatment with various doses of H_2_O_2_ for 1 h. The level of Nrf2 or DHX9 protein were measured by Western blot with Vinculin as a loading control. Quantification of Nrf2 band intensities by ImageJ is shown as means ± SD from three independent experiments (*B* and *D*). RNA was extracted from total collection of ribosomes for detection of Nrf2 mRNA by RT-qPCR with GAPDH as a loading control (*E*). The level of Nrf2 transcript was calculated as 2^-ΔCT^ over that of GAPDH and shown as means ± SD of triplicates of one experiment representative of three (*E*). ∗ indicates *p* < 0.05 compared to the control group by student's *t* test (*B* and *D*) or one-way ANOVA (*E*). DHX9, DExH-box helicase-9; qPCR, quantitative PCR.

To determine whether or not DHX9 mediates *de novo* Nrf2 protein translation, we used an siRNA to knock down DHX9 protein. With decreased levels of DHX9 protein, H_2_O_2_-induced Nrf2 elevation was inhibited (Fig. 3, *A* and *B*). DHX9 is a helicase and its activity can be inhibited by YK-4-279 (27). We found that YK-4-279 was effective in preventing H_2_O_2_ from inducing Nrf2 protein elevation (Fig. 3, *C* and *D*).

Since DHX9 is known as a nuclear protein, whereas protein translation occurs in the cytoplasm, we asked whether oxidative stress caused an export of Nrf2 mRNA and whether the DHX9 inhibitor blocked such nuclear export. No increase of Nrf2 mRNA was observed among total RNA (supplemental Fig. S1*A*), consistent with the lack of transcriptional activation of Nrf2 gene as reported previously with different cell types (9, 10, 26). The measurements of Nrf2 mRNA levels in the nuclear or cytosolic fractions did not reveal nuclear export of Nrf2 mRNA due to H_2_O_2_ treatment (supplemental Fig. S1, *B* and *C*). An increase of Nrf2 mRNA in the ribosomes was observed, consistent with *de novo* Nrf2 protein translation by H_2_O_2_ treatment (Fig. 3*E*). YK-4-279 was able to block Nrf2 mRNA increase in the ribosome by H_2_O_2_ treatment (Fig. 3*E*). This suggests that the helicase activity of DHX9 is important for Nrf2 mRNA association with the ribosomes in order for *de novo* Nrf2 protein translation to occur.

### An increased DHX9 association with ribosomes by H_2_O_2_ treatment

**Fig. 4:**
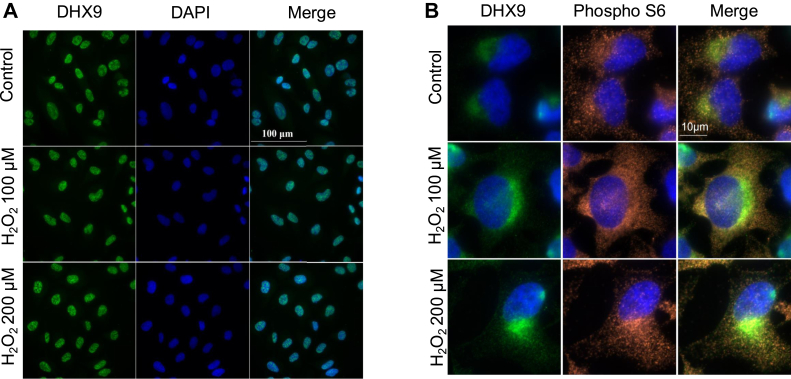
**Nuclear localization of DHX9.** HeLa cells at a confluency of 50% were treated with 0, 100, or 200 μM H_2_O_2_ for 1 h. After fixing with 4% paraformaldehyde, cells were permeabilized by either 0.2% Triton X-100 (*A*) or 0.5% saponin (*B*). DHX9 was detected by immunocytochemistry with secondary antibody conjugated with Alexa Fluor 488 (*green*) and nuclei counter stain with 4′,6-diamidino-2-phenylindole (*blue*). Phospho S6 was recognized by specific primary antibody and Alexa Fluor 568–conjugated secondary antibody (*red*). DHX9, DExH-box helicase-9.

DHX9 is known as a nuclear protein (28, 29). Immunocytochemistry staining indeed revealed the nuclear localization of DHX9 (Fig. 4*A*). Quantification of nuclear signal of DHX9 using ImageJ software (https://imagej.net) revealed an average intensity of 111927 ± 17002, 111104 ± 18459, or 90106 ± 21988 (reduced compared to 0 μM H_2_O_2,_ *p* < 0.05) for 10 randomly chosen cells in 0, 150 μM or 300 μM H_2_O_2_-treated group, respectively, indicating a detectable reduction of nuclear DHX9 at 300 μM H_2_O_2_. Using a technique to detect DHX9 protein only in the cytosol, it appeared that DHX9 was enriched in the perinuclear area (Fig. 4*B*). We stained the cells with phospho S6 protein to address whether DHX9 colocalized with active ribosomes (Fig. 4*B*). The Pearsons correlation coefficient values were 0.38 ± 0.09, 0.43 ± 0.08 or 0.45 ± 0.09, for 0, 100 μM H_2_O_2_, or 200 μM H_2_O_2_-treated cells, indicating no colocalization. Mander’s coefficients also did not support colocalization of DHX9 with phospho S6.

**Fig. 5:**
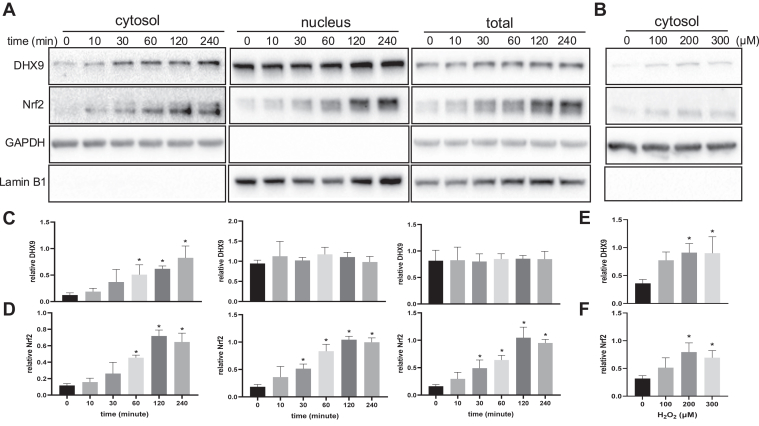
**Increases of cytosolic DHX9 protein by H_2_O_2_ treatment.** HeLa cells were treated with 100 μM H_2_O_2_ for 10 min and cultured in freshly replaced DMEM containing 0.5% FBS for indicated time (*A*) or with various doses of H_2_O_2_ for 1 h (*B*) before subcellular fractionation. The levels of DHX9 protein in the cytosol, nuclear fraction, or total cell lysate were detected by Western blot using Nrf2 protein as a comparison. Lamin B1 was used as a marker of nuclear fraction and GAPDH was used as a loading control for the cytosol or total cell lysates. The band intensities of DHX9 (*C* and *E*) or Nrf2 (*D* and *F*) were quantified and normalized to Lamin B1 or GAPDH. The data represent means ± SD from three independent experiments (*C*–*F*). ∗ indicates *p* < 0.05 compared to the control group from the same fraction by one-way ANOVA. DHX9, DExH-box helicase-9; DMEM, Dulbecco's modified Eagle's medium; FBS, fetal bovine serum.

We found that H_2_O_2_ treatment caused an increase of DHX9 protein in the cytosol (Fig. 5, *A* and *B*). The time-course and dose-response studies revealed that the cytosolic DHX9 increase correlated with elevation of Nrf2 protein in the cytoplasm (Fig. 5). No significant changes of DHX9 level were observed in total cell lysates or the nuclear fraction (Fig. 5).

**Fig. 6:**
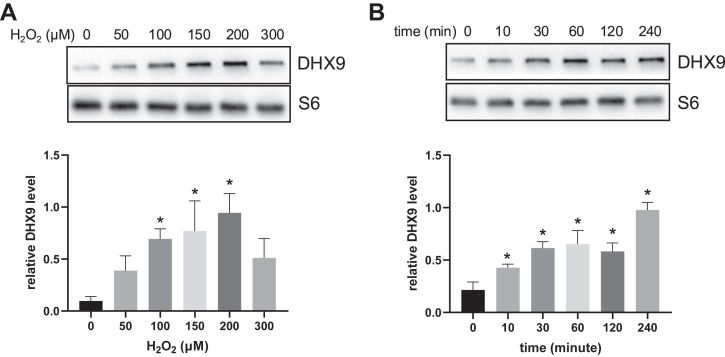
**DHX9 increased the association with ribosomes due to H_2_O_2_ treatment.** HeLa cells were treated with an indicated dose of H_2_O_2_ for 1 h (*A*) or with 100 μM H_2_O_2_ for 10 min for collection at indicated time points (*B*). Total ribosomes were collected after loading cytosolic fractions to 10% + 35% sucrose cushion for 2-h ultracentrifugation at 32,000 rpm with a SW41Ti rotor. The isolated ribosomes were denatured in 1× Laemmli buffer for measurements of DHX9 protein by Western blot. Quantification of band intensities is shown as means ± SD from three independent experiments. ∗ indicates *p* < 0.05 compared to the control 0 μM or 0 min H_2_O_2_ treatment group by one-way ANOVA. DHX9, DExH-box helicase-9.

To determine whether DHX9 becomes associated with translation machinery, we measured the level of DHX9 protein in total collection of ribosomes. A clear increase of DHX9 in total ribosome collections was observed in a H_2_O_2_ dose-dependent manner (Fig. 6*A*), detectable 10 min after H_2_O_2_ treatment (Fig. 6*B*).

**Fig. 7:**
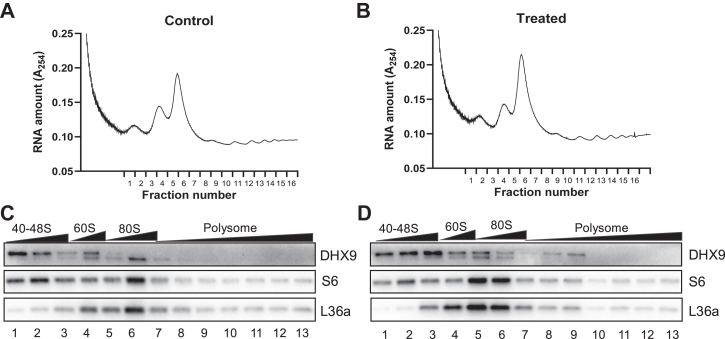
**DHX9 distribution in ribosomal fractions.** Control or 100 μM H_2_O_2_-treated HeLa cells were harvested at 1 h for collection of cytosolic fractions, which were loaded for ultracentrifugation in 10% to 50% linear sucrose gradient. The samples were subjected to liquid chromatography at a flow speed of 1 ml/min with UV absorbance of 254 nm monitored and recorded as shown (*A* and *B*). The fractions were collected every 0.5 min for measuring the distribution of DHX9 protein in control (*C*) or H_2_O_2_-treated groups (*D*) by Western blot. S6 serves as a marker of ribosomal small subunit and L36a was a marker for ribosomal large subunit. DHX9, DExH-box helicase-9.

The ribosomes were fractionated into the small or large subunit, monosome or polysome to address which fraction is associated with DHX9. We observed that the level of DHX9 protein increased due to H_2_O_2_ treatment in the fractions corresponding to the second half of 40-48S ribosomal peak (lane 2, Fig. 7, *C* and *D*) and in the fraction corresponding to the gap between 40 to 60S ribosomal peak (Lane 3, Fig. 7, *C* and *D*). Judging from the presence of S6 and L36a in this fraction, it suggests that this fraction contains both small and large ribosomal subunit. The lighter 80S monosomes (lane 5) and polysomes (lane 8–9) also showed an increase of DHX9 in cells treated with H_2_O_2_ (Fig. 7). This suggests a possible role of DHX9 in the formation of 48S initiation complex (IC) and 80S IC, and in translation initiation as well as translation elongation.

**Fig. 8:**
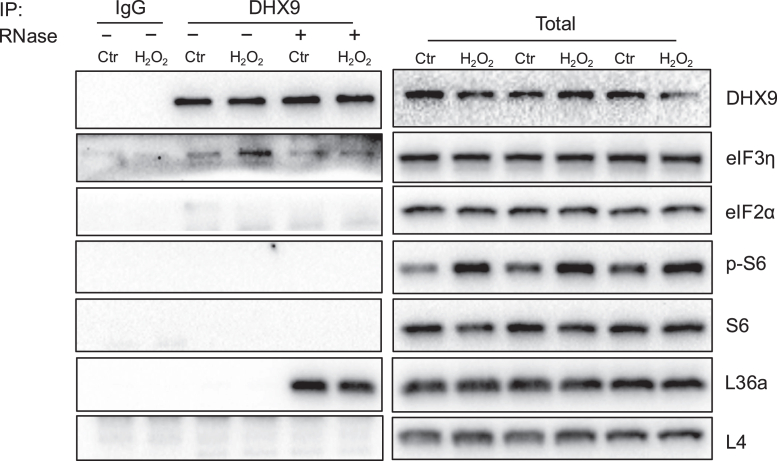
**DHX9 interacts with eIF3η in an RNA-dependent manner.** HeLa cells were treated with or without 100 μM H_2_O_2_ for 1 h and lysed in RIPA buffer. RNase inhibitor or RNase A/T1 was added to the cell lysates of indicated groups. Cell lysates were incubated with DHX9 antibody or IgG followed by protein A/G agarose beads to isolate the coimmunoprecipitated complex. The proteins in the complex were eluted for analysis by Western blot. DHX9, DExH-box helicase-9; RIPA, radioimmunoprecipitation.

Translation initiation requires recognition of the target mRNA strand by binding of eIF4F complex containing eIF4E, eIF4G, and eIF4A (3, 30, 31). This mRNA–eIF4F complex becomes available for landing of 43S PIC to form 48S IC, containing eIF1, eIF1A, eIF3, eIF2/GTP/tRNA_i_^Met^ ternary complex, and the 40S small subunit of the ribosome. We performed co-IP experiments to investigate the translation initiation factors that bind to DHX9. There was no physical binding of DHX9 with phospho S6 or total S6 (Fig. 8). Similarly, there is a lack of physical interaction of DHX9 with L36a or L4 of ribosomal large subunit protein (Fig. 8), except that DHX9 gained the interaction with L36a following RNase digestion for a reason unknown.

We found that DHX9 can bind to eIF3η, but not eIF2α (Fig. 8). H_2_O_2_ treatment caused an increased association of DHX9 with eIF3η, which was sensitive to RNase digestion (Fig. 8). This suggests that the gain of DHX9 interaction with eIF3η is RNA binding–dependent during oxidative stress.

### Possible Negative Regulator for DHX9 Binding to Nrf2 5′UTR or Ribosomes

**Fig. 9:**
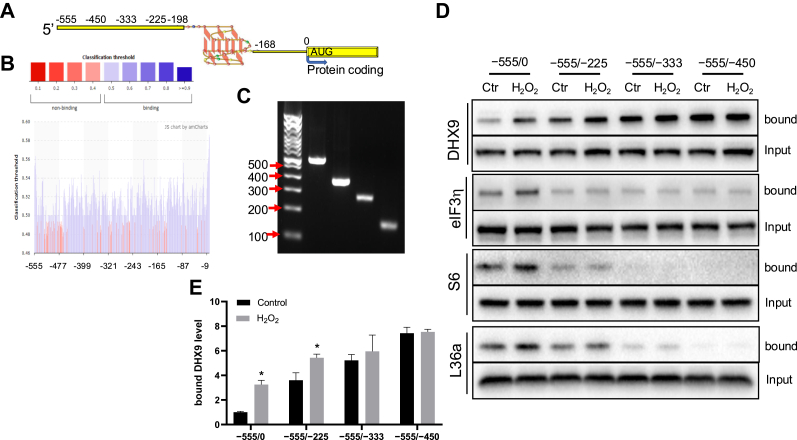
**DHX9 binding site in Nrf2 5′UTR.** Schematic diagram shows Nrf2 5′UTR containing G-quadruplex (*A*). The predicted contact sites for DHX9 protein are shown along Nrf2 5′UTR sequence, per PRIdictor (http://bclab.inha.ac.kr/pridictor/) (*B*). The DNA templates with T7 promoter at the 5′ end is confirmed by an agarose gel electrophoresis for *in vitro* transcription of each of Nrf2 5′UTR fragments (*C*). Full length (−555/0) or fragments (−555/−225, −555/−333, −555/−450) of biotinylated Nrf2 5′UTR RNA (50 pmol) were incubated with cytosolic extracts from HeLa cells harvested at 1 h with or without 100 μM H_2_O_2_ treatment. Biotinylated RNA and its binding proteins were pull down by streptavidin agarose beads for detection of the proteins by Western blot (*D*). Quantification of bound DHX9 is shown as means ± SD from three independent experiments (*E*). ∗ indicates *p* < 0.05 compared to the control group with the same RNA fragment for pulldown by student's *t* test (*E*). DHX9, DExH-box helicase-9.

Since DHX9 was discovered as a protein binding to 555 nt Nrf2 5′UTR, we used a computation tool, PRIdictor (http://bclab.inha.ac.kr/pridictor/) (32), to predict the specificity of DHX9 binding site on Nrf2 5′UTR. Nrf2 5′UTR contains a G-quadruplex structure in −168 to −198 nt region (Fig. 9*A*). There is evidence of DHX9 binding to and unwinding G-quadruplex structures (33). By inputting DHX9 protein sequence and the sequence of Nrf2 5′UTR, the PRIdictor software predicted that DHX9 might have weak binding to most of the nucleotides in Nrf2 5′UTR, instead of G-quadruplex region (Fig. 9*B*). In an effect to test DHX9 binding sites, we generated RNA fragments containing sequence deletions (Fig. 9*C*). In the sequence deletion experiment, the −555/−225 nt fragment does not contain the G-quadruplex but was able to gain DHX9 binding with the lysates from H_2_O_2_-treated cells (Fig. 9*D*). Interestingly, the baseline DHX9 binding increased with the reduction of Nrf2 5′UTR sequence and H_2_O_2_ treatment did not increase the binding for −555/−333 or −555/−450 fragments (Fig. 9*D*). This suggests the possibility of a negative element in −333 to 0 nt region, which prohibits the baseline DHX9 binding, and H_2_O_2_ treatment was able to overcome such negative element for DHX9 binding.

**Fig. 10:**
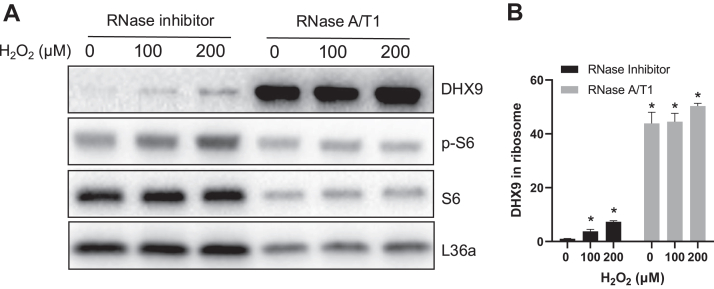
**DHX9 associates with ribosomes independent of RNA binding.** HeLa cells treated with indicated doses of H_2_O_2_ for 1 h before lysis in the presence of RNase inhibitor or RNase A/T1. Total ribosomes were isolated by 10% + 35% sucrose cushion and 2-h ultracentrifugation at 32,000 rpm with a SW41Ti rotor from cell lysates for Western blot (*A*). DHX9 bands were quantified by ImageJ and presented as means ± SD from three independent experiments (*B*). ∗ indicates *p* < 0.05 compared to the 0 μM H_2_O_2_ group with RNase inhibitor by one-way ANOVA. DHX9, DExH-box helicase-9.

To test whether the negative element is an RNA, we performed RNase digestion experiments. If such negative element is an RNA, for example the −330 to 0 nt region of Nrf2 5′UTR, RNase digestion is expected to remove the element and enhance DHX9 association with the ribosomal complex. Ribosomal preparation from cell lysates showed an increased DHX9 association with the ribosomes following RNase treatment (Fig. 10). Therefore, the data support the presence of a negative element in RNA and that H_2_O_2_ treatment was able to overcome such negative element for DHX9 binding to Nrf2 5′UTR and DHX9 association with ribosomes.

## Discussion

In an effort to understand how Nrf2 protein is translated when cells encounter oxidative stress, we discovered DHX9 as a binding protein for Nrf2 5′UTR by mass spectrometry. Validations using Far Western blot or ribonucleoprotein IP showed that oxidative stress indeed caused an elevated DHX9 binding to Nrf2 mRNA *in vitro* and *in cellulo*. The level of DHX9 protein increased in the cytoplasm in correlation with its gain of ribosomal association and with increased level of Nrf2 protein. IP experiments revealed DHX9 increased interaction with eIF3η in an RNA-dependent manner due to H_2_O_2_ treatment. Knocking out DHX9 expression using siRNA or inhibition of DHX9 activity was able to prevent H_2_O_2_ from inducing Nrf2 mRNA association with the ribosomes or Nrf2 protein elevation. Our data suggest a scenario in which oxidants cause an increased DHX9 binding to Nrf2 5′UTR, and this binding contributes the interaction with 43S PIC containing eIF3η for subsequently assembly of 48S IC and 80S IC in order to initate Nrf2 protein translation.

DHX9 is an ATP-dependent DExH RNA helicase, also known as the RNA helicase A (34, 35, 36, 37). The human genome contains 64 RNA helicases and 31 DNA helicases (38). Unlike a classical DNA helicase, which cuts, unwind, and sometimes translocates one strand of the substrate DNA helix, the function of RNA helicases can be diverse. As a result, RNA helicases participate in regulations of transcription, RNA metabolism and protein translation. Whereas destabilizing or unwinding dsRNA represents an important character of RNA helicase function, many RNA helicases act as a chaperone for folding RNA into a specific secondary or tertiary structure, or for revamping of RNP complexes (39). The DExH RNA helicases exhibit the ability to cut and translocate an RNA strand, altering its secondary or tertiary structure (35). In addition to unwinding of dsRNAs, DExH family members are capable of remodeling of RNP complex, disassembling spliceosomes and assembling ribosomes (37).

IRES-mediated protein translation was first discovered with viral proteins when an infection caused an inhibition of cellular protein synthesis (17, 40). DHX9 has been reported to interact with Chikungunya viral RNA for the viral protein translation (41). With *in vitro* assays for RNA binding and protein translation, it was found that the N-terminal domain of DHX9 protein was capable of recognizing target mRNA strand and tethering the ATP-dependent helicase to the 5′UTR for activation of translation (42). In the 5′UTR of type I collagen gene, DHX9 binding results from its interaction with La ribonucleoprotein domain family member 6. Although H_2_O_2_ induced La interaction with Nrf2 5′UTR (8), we did not observe DHX9 interaction with La (data not shown). Nevertheless, the small molecular DHX9 inhibitor YK-4-279 complements with DHX9 siRNA experiment in demonstrating the requirement of DHX9 for Nrf2 protein induction. YK-4-279 was first identified as an inhibitor of DHX9 by binding to EWS-FLI, a cofactor of DHX9. YK-4-279 has ongoing clinical trials for Ewing sarcoma as an effective inhibitor of cell proliferation (27). Investigation on the mechanism of YK-4-279 action has led to the finding that YK-4-279 does not affect RNA binding affinity of DHX9 but causing an altered RNA-binding profile (27). This suggests the possibility that DHX9 binding to Nrf2 5′UTR is shifted by YK-4-279 or EWS-FLI1 may act as a cofactor of DHX9 for Nrf2 protein translation regulation.

One possibility that cannot be excluded from our data is that Nrf2 5′UTR may interact with an RNA element, and DHX9 binds to such complex for removal the inhibitory effect of the RNA element. Although removal of an inhibitory RNA element provides a simplistic explanation, the enhanced association of DHX9 with ribosomes due to RNase A/T1 digestion, which cleaves ssRNA, could result from structure elements in the RNA that preclude RNase A/T1 degradation such as dsRNA. Therefore, how DHX9 affects Nrf2 protein translation under stress condition remains to be fully elucidated.

How oxidative stress activates DHX9 remains unknown. DExH family of RNA helicases share a conserved helicase core consisting of RecA1 and RecA2 domains for ATP binding, ATP hydrolysis, and RNA interaction (37). The C-terminal region flanks the winged helix, Ratchet-like and oligosaccharide-binding fold domains, for interaction with the helicase core. This suggests that the C-terminal domain serves as an inhibitor by locking up the enzyme in its inactive conformation. The oligosaccharide-binding fold interacts with G-patch domain containing proteins to convert the two-domain helicase core into a ring-like conformation for ATP and substrate binding, serving to activate the enzyme (36). A family of glycine rich and 40 to 50 amino acids long G-patch domain containing proteins have been reported from the yeast *Saccharomyces cerevisiae* (36). The homologs of these G-patch domain–containing proteins have been discovered in the humans. In addition to G-patch domain containing proteins, DHX9 protein contains 1270 amino acids and multiple sites for potential posttranslational modifications, from phosphorylation to acetylation or sumoylation (www.uniprot.org). The observed molecular weight differences of DHX9 in 60-80S ribosomes (Fig. 8) and the presence of sumoylation consensus sequences LKAE/LKNE in DHX9 protein point to possible sumoylation (43). Therefore, in addition to possible interactions with G-patch domain–containing proteins, DHX9 may undergo posttranslational modifications for its activation.

Our earlier study revealed that nuclear to cytoplasmic shuttling of RNPs plays a role in *de novo* Nrf2 protein translation (8). Leptomycin B inhibited Nrf2 induction and nuclear export of La to the cytosol for increased Nrf2 5′UTR binding and ribosomal association (8). Like La, an increase of cytosolic DHX9 was observed with H_2_O_2_ treatment (Fig. 5). Since DHX9 is a nuclear protein, an increased DHX9 in the cytosol is likely derived from nuclear exportation. Because the majority of DHX9 are located in the nuclei, and the amount of DHX9 in the cytosol is low, current technique for measuring protein levels, i.e., Western blot, is not capable of displaying small reduction in the nuclear fraction. Nevertheless, the data from our immunocytochemistry experiments point to a decrease of nuclear DHX9 protein with 300 μM H_2_O_2_ treatment, providing an explanation for its increase in the cytoplasm for regulating Nrf2 protein translation.

We have discovered that DHX9 increases its interaction with eIF3η due to H_2_O_2_ treatment. Such interaction is consistent with DHX9 increasing its interaction with Nrf2 mRNA and its distribution in the 40-48S fraction of ribosomes. Hartman, *et al.* reported that DHX9 interaction with a structured control element in 5′UTR of a retrovirus or from cellular JunD gene facilitates translation initiation (44). The control element in Nrf2 5′UTR for DHX9 binding and translation initiation remains to be discovered. Nrf2 5′UTR fragment binding assays revealed negative elements in −330 to 0 nt region prohibiting the baseline DHX9 interaction. This region recruits the ribosomes (Fig. 9, the presence of S6 in Nrf2 5′UTR binding complex) and is therefore important for DHX9 engagement in translation initiation of Nrf2 protein. Revealing the control elements in this region will provide the mechanistic basis for DHX9 action.

## Data Availability

The raw mass spectrometry data are available *via* ProteomeXchange with identifier PXD055838.

All methods and data reported in the article are available upon request.

## Supplemental data

This article contains supplemental data.

## Conflict of interest

The authors declare no competing interests.

## Supplementary Data

Supplement Table 1

SupplementTable 2

SupplementFig 1
