# Supplementary material for: DExH-Box Helicase 9 Participates in De Novo Nrf2 Protein Translation Under Oxidative Stress
Source: Mol Cell Proteomics. 2025 Apr 23;24(6):100977. doi: 10.1016/j.mcpro.2025.100977 (PMC12166428; doi:10.1016/j.mcpro.2025.100977)
Supplement: SupplementFig 1 [file mmc3.pdf]

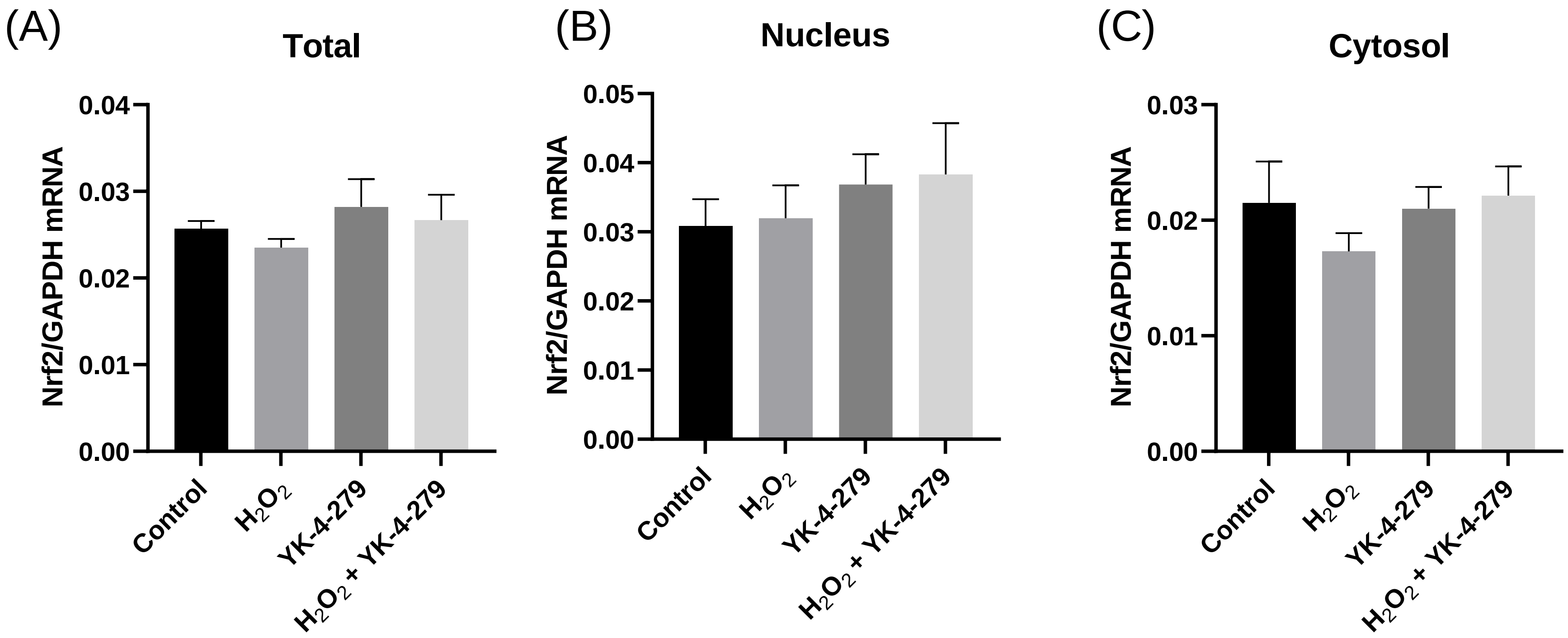

Nrf2 mRNA Does Not Translocate from Nuclei to Cytosol. HeLa cells were pre-treated with 1 μM YK-4-279 for 16 hours before 1 hr treatment with 100 μM H_2O_2. Total RNA was extracted from total cell lysate (A), nuclear fraction (B), cytosolic fraction (C) for detection of Nrf2 mRNA by RT-qPCR with GAPDH as a loading control. The level of Nrf2 transcript was calculated as $2^{-\Delta CT}$ over that of GAPDH, and shown as means ± SD of triplicates of one experiment representative of three. * indicates $p<0.05$ compared to the control group by one-way ANOVA.
